# Neurological Impacts of Chronic Methylmercury Exposure in Munduruku Indigenous Adults: Somatosensory, Motor, and Cognitive Abnormalities

**DOI:** 10.3390/ijerph181910270

**Published:** 2021-09-29

**Authors:** Rogério Adas Ayres de Oliveira, Bruna Duarte Pinto, Bruno Hojo Rebouças, Daniel Ciampi de Andrade, Ana Claudia Santiago de Vasconcellos, Paulo Cesar Basta

**Affiliations:** 1 Centro de Dor, Departamento de Neurologia, Hospital das Clínicas, Faculdade de Medicina, Universidade de São Paulo (USP), São Paulo 05403-000, Brazil; roger.adas.doc@gmail.com (R.A.A.d.O.); brunaduartencl@gmail.com (B.D.P.); bruhojo@hotmail.com (B.H.R.); ciampi@usp.br (D.C.d.A.); 2 Laboratório de Educação Profissional em Vigilância em Saúde, Escola Politécnica de Saúde Joaquim Venâncio, Fundação Oswaldo Cruz (EPSJV/Fiocruz), Rio de Janeiro 21040-900, Brazil; anacsvasconcellos@gmail.com; 3 Departamento de Endemias Samuel Pessoa, Escola Nacional de Saúde Pública, Fundação Oswaldo Cruz (ENSP/Fiocruz), Rio de Janeiro 21041-210, Brazil

**Keywords:** mercury exposure, neurological abnormalities, indigenous people, Amazon, environmental impacts, illegal mining

## Abstract

There has been increasing evidence about mercury (Hg) contamination in traditional populations from the Amazon Basin due to illegal gold mining. The most concerning health impact is neurotoxicity caused by Hg in its organic form: methylmercury (MeHg). However, the severity and extent of the neurotoxic effects resulting from chronic environmental exposure to MeHg are still unclear. We conducted a clinical-epidemiological study to evaluate the neurological impacts of chronic MeHg exposure in Munduruku indigenous people, focusing on somatosensory, motor, and cognitive abnormalities. All participants were subjected to a systemized neurological exam protocol, including Brief Cognitive Screening Battery (BCSB), verbal fluency test, and Stick Design Test. After the examination, hair samples were collected to determine MeHg levels. Data collection took place between 29 October and 9 November 2019, in three villages (*Sawré Muybu*, *Poxo Muybu*, and *Sawré Aboy*) from *Sawré Muybu* Indigenous Land, Southwest of Pará state. One hundred and ten individuals >12 years old were included, 58 of which were men (52.7%), with an average age of 27.6 years (range from 12 to 72). Participants’ median MeHg level was 7.4 µg/g (average: 8.7; S.D: 4.5; range: 2.0–22.8). In *Sawré Aboy* village, the median MeHg level was higher (12.5 µg/g) than in the others, showing a significant statistical exposure gradient (Kruskal–Wallis test with *p*-value < 0.001). Cerebellar ataxia was observed in two participants with MeHg levels of 11.68 and 15.68 µg/g. Individuals with MeHg exposure level ≥10 µg/g presented around two-fold higher chances of cognitive deficits (RP: 2.2; CI 95%: 1.13–4.26) in BCSB, and in the verbal fluency test (RP: 2.0; CI 95%: 1.18–3.35). Furthermore, adolescents of 12 to 19 years presented three-fold higher chances of verbal development deficits, according to the fluency test (RP: 3.2; CI 95%: 1.06–9.42), than individuals of 20 to 24 years. The worsened motor and cognitive functions are suggestive of neurotoxicity due to chronic MeHg exposure. In conclusion, we believe monitoring and follow-up measures are necessary for chronic mercury exposed vulnerable people, and a basic care protocol should be established for contaminated people in the Brazilian Unified Health System.

## 1. Introduction

Methylmercury (MeHg) is a toxic organo-mercurial compound which acts systemically in the human body [1,2]. The intake dose, exposure duration, and frequency, in addition to the exposure route and age of the individual (or stage of development), are factors that determine the extent of damage caused by MeHg and which organs or systems are worst affected [3].

After the Minamata and Niigata disasters in Japan in the 1950s and 1960s, the international scientific community agreed that methylmercury’s main target is the Central Nervous System (CNS) [4]. During that time, it was observed that adult individuals who regularly consumed contaminated fish presented neurological abnormalities such as paresthesia, ataxia, dysarthria, visual field alterations, and hearing impairments. In addition, these events showed that the prenatal period is the most vulnerable stage to the deleterious effects of MeHg. Many women who were exposed during pregnancy gave birth to children with severe congenital malformations and significant cognitive impairments [5,6,7]. Years later, the clinical manifestation caused by the exposure to high MeHg levels during adult life or pregnancy became known as Minamata Disease [8,9].

The MeHg intoxication episode in Iraq, in the early 1970s, was even more devastating than Japan’s tragedy. Because it was an acute exposure episode, there were more than 6000 hospitalizations due to intoxication and hundreds of deaths in a short period of time [10,11]. In that time, the refinement of the spectrometry of atomic absorption technique for analyzing Hg in human tissue made laboratorial measurements more precise. Consequently, it was possible to establish a consistent relationship between the signs and symptoms presented by the exposed individuals and mercury concentration in exposure biomarkers (e.g., hair and blood) [6]. Based on scientific evidence, the World Health Organization established [4] that the neurological effects of MeHg exposure can be found in 5% of adult individuals who ingest 3 to 7 µg daily of MeHg per kilogram of body weight (kg bw). This daily intake dose corresponds to levels of 200 µg/L in blood or 50 µg/g in hair. However, these indexes are constantly revised, and some are updated as a result of new technologies and current research carried out in different parts of the world.

Since the 1990s, research conducted in the Amazon region identified adult individuals with neurological abnormalities probably associated with the consumption of contaminated fish. In those individuals, levels of methylmercury in hair samples varied between 10 and 20 µg/g [12,13,14,15,16,17]. Similar findings were reported in riparian populations in Bolivia and Suriname [18,19].

It is worth noting that the peculiar characteristics of the Amazon’s ecosystem and the social dynamics of its traditional populations may be responsible for the different thresholds for the onset of neurological effects. In that region, the majority of the mercury human exposure is due to artisanal small-scale gold mining (ASGM) [20]. ASGM causes the liberation of large quantities of Hg in the ecosystem and, at the same time, mobilizes natural Hg present in the soil. In addition to ASGM, the construction of hydroelectric plants and dams, deforestation, and fires also alter the Hg cycle in the region, increasing the risk of human contamination [20,21,22,23,24]. Moreover, the traditional people living there are considered to be among the largest consumers of fish in the world [25,26,27].

In the aquatic environment, the Hg released by the ASGM suffers methylation, mediated by microorganisms, transforming it into methylmercury (MeHg). Most of the toxicity attributed to MeHg is due to its biomagnification capacity in aquatic trophic chains and to the ability to overcome blood–brain and placenta barriers. Because fish consumption is the main protein source in many traditional populations in the Amazon, chronic exposure to high levels of mercury can cause various negative consequences and create health problems in local populations [20,28,29,30,31,32]. After intake of contaminated fish, almost all MeHg is quickly absorbed by the gastrointestinal tract. The absorbed MeHg forms a conjugate with the cysteine molecules present in the blood and, afterward, is distributed to virtually all human body tissue. When the MeHg reaches the CNS, it undergoes oxidation and tends to accumulate, leading to potentially irreversible cerebral tissue damage [33]. 

Experimental evidence shows that MeHg neurotoxicity is marked by an increase in oxidative stress, by the presence of mitochondrial lesions, by the release of excitatory amino acids, and by proteomic expression alterations, which can lead to cellular dysfunction and death [34,35,36]. Furthermore, the chronic exposure to high levels of MeHg has been associated with memory and learning deficits, motor function abnormalities, hearing loss, and a reduced visual field [3,20,37,38,39,40,41,42]. 

In the Amazon region, few clinical studies have correlated MeHg exposure levels with neurological symptoms [12,13,14,16,17]. Nevertheless, none of these studies included indigenous populations. Therefore, there is insufficient evidence about neurotoxicity concerning indigenous populations chronically exposed to MeHg in the Amazon Basin.

With the lack of evidence on this topic in mind, this study aimed to describe neurological clinical manifestations, focusing on somatosensory, motor, and cognitive abnormalities, in adults indigenous from three Munduruku villages in the Amazon Basin. The ultimate goal was to explore possible associations between neurological abnormalities with chronic methylmercury exposure.

## 2. Materials and Methods

### 2.1. Study Design and Field Work

We conducted a cross-sectional clinical-epidemiological study including indigenous people older than 12 years old living in three villages: *Sawré Muybu* (SM), *Poxo Muybu* (PM), and *Sawré Aboy* (SA), in the *Sawré Muybu* Indigenous Land (IL), located at Middle-Tapajós River, in the southwest region of Pará state, Brazilian Amazon.

Data collection occurred between 29 October and 9 November 2019, according to a multidisciplinary and inter-institutional study developed to clarify the consequences and assess the impacts of illegal mining in the *Sawré Muybu* IL. For additional information, see Basta et al. [43].

### 2.2. Evaluated Population

We performed a census in the three villages mentioned above, and all indigenous individuals aged 12 or older were invited to participate in the study. Therefore, probabilistic sampling methods were not used to select participants. A 15-year-old man from *Sawré Muybu* village, who had cerebral palsy, was excluded from the study. 

#### Fish Consumption Estimates

Like the majority of the traditional groups living in the Amazon Basin, Munduruku people consume large quantities of fish. Recently, Vasconcellos et al. [44] carried out an investigation to estimate the fish consumption of the Munduruku indigenous people living in the study area and compared their findings with the acceptable levels proposed by the FAO/WHO [45] and by the U.S.EPA [46]. 

During one week, the authors collected 88 fish specimens representing 17 species and four trophic levels to analyze the total mercury (THg) concentration in muscle tissue. They also developed an empirical method to estimate fish consumption from catch effort. The average daily fish consumption estimated for adult men was 216.75 g, and for women of childbearing age was 168.58 g. Considering that about 90–95% of total mercury species detected in fish samples is in the form of MeHg [47,48,49], we assume that all of the Hg detected in the fish samples is compounded by MeHg.

The average MeHg levels in samples of non-piscivorous fish (n = 57) was 0.10 μg/g (SD = 0.09) and the average for piscivorous fish (n = 31) was 0.44 μg/g (SD = 0.34). The highest level of methylmercury in muscle tissue (1.95 μg/g) was observed in the *Serrasalmus rhombeus* (also known as *piranha preta* in Portuguese), a fish not only located at the top of the food chain, but also highly appreciated and regularly consumed by the Munduruku [44]. 

Moreover, the authors devised four scenarios to estimate the MeHg exposure, considering the fish consumption from catch effort: (i) in the rainy season; (ii) in the dry season; (iii) based on the weighted average of medium mercury levels detected in piscivorous and non-piscivorous species; and (iv) based on the 95^th^ percentile of mercury concentrations in piscivorous and non-piscivorous species (1.42 and 0.29 μg/g, respectively). 

According to the authors, scenario (iii) most closely matches the local reality, in which the estimates of MeHg ingestion for Munduruku adult men ranged from 1.84- to 8.28-fold above the safe daily methylmercury intake recommended by FAO/WHO [45] and U.S.EPA [46], respectively. The estimates ranged from 3.17- to 7.29-fold above the FAO/WHO [45] and U.S.EPA [46], respectively, for women of childbearing age.

Summarizing, the studied Munduruku villages are at severe risk of harm due to the ingestion of MeHg-contaminated fish.

### 2.3. Neurological Examination

All participants were submitted to a systemized neurological examination protocol, specifically developed for this research, to make the evaluation feasible in the adverse conditions of the fieldwork in the villages. The evaluations were conducted by neurologists (BDP, BHR, and RAAO) familiar with clinical neurological semiology and duly trained to apply the protocol and use the examining instruments.

The explanations about the clinical procedures for the neurological exam were verbally presented to the participants in Brazilian Portuguese. When necessary, for indigenous individuals that had difficulties with Portuguese, an indigenous translator (indigenous health agents, teachers, or chiefs) from the village would assist.

#### 2.3.1. Static Balance and Walking Evaluation

The evaluation of static balance and walking was performed by visual inspection. Participants were asked to remain in an orthostatic position to perform the Romberg test, with eyes open and closed, sequentially. Then, participants were asked to walk towards the examiner.

#### 2.3.2. Cranial Nerve Examination

The following parameters were evaluated: visual campimetry by confrontation, pupil reflex and external ocular motricity, facial motricity and symmetry, facial sensibility, palate elevation, gag reflex and tongue motricity.

#### 2.3.3. Sensory Testing

The following instruments were used to evaluate the respective somatosensory modalities:

(a) Sharp nickel-plated pin (Bacchi^®^ number 29): mechanical nociceptive perception.
(b) Sorri Bauru esthesiometer/Von Frey 10g monofilament: tactile sensitivity.
(c) Dry cotton wad: dynamic tactile sensitivity.
(d) 128 Hz diapason: tactile vibration sensitivity and thermic sensitivity to cold [50].

The sensitivity was tested in upper and lower limbs at three distinct points for each sensory modality, in the proximal and distal segments. The sensitivity was classified as normal (0) or abnormal (1) depending on the abnormality occurrence in at least one parameter.

For the clinical diagnosis of distal symmetric polyneuropathy, criteria from the American Academy of Neurology were used, which consider the occurrence of distal sensory deficits, in addition to the clinical signs of neuropathy, defined as the presence of abnormalities in at least one somatic sensory domain and/or alterations in the ankle jerk reflex [51].

#### 2.3.4. Motor Function

Muscular strength was evaluated through maneuvers against resistance in all four limbs, on proximal and distal segments. Muscular hypertonia and bradykinesia were evaluated through passive mobilization of the four members, through finger tapping maneuvers, facial expression mimicking, and spontaneous mobilization. Coordination was evaluated through finger to nose, heel to knee, and diadochokinesis (ability to make alternating quick movements) tests. The deep osteotendinous ankle jerk reflex was tested with the Babinski hammer, according to the Hallett [52] myotatic reflex score. Toe amyotrophy was investigated through inspection.

#### 2.3.5. Cognitive Evaluation

All individuals were submitted to a cognitive evaluation by the authors (BDP, BHR and RAAO), immediately after the neurological exam, as part of the evaluation protocol.

An indigenous translator from the village was present in the interviews to help with the communication with indigenous individuals who had difficulties with Portuguese.

The Brief Cognitive Screening Battery (BCSB) [53], the verbal fluency test in the animal category [54], and the stick design test [55] were used as instruments of cognitive evaluation. These instruments were selected given their appropriateness for populations with low levels of education and for the easy application in adverse conditions in the field.

##### Brief Cognitive Screening Battery (BCSB)

The BCSB is an instrument developed by the Cognitive and Behavior Neurology Group of the *Hospital das Clínicas* of the Medical School of the *Universidade de São Paulo* [53], which entails naming and remembering 10 drawings of simple and known objects in black and white presented in a single picture. This instrument evaluates, in sequence, the following domains: (a) visual perception and naming, (b) incidental memory, (c) immediate memory, (d) learning, (e) verbal fluency test (described below), (f) 5 min belated memory, and g) recognition.

The following cutoffs were used in the tests to evaluate the domains’ normality: immediate memory (≥5), learning (≥7), verbal fluency (≥9), and delayed recall (≥6). Alteration in at least one of these domains was considered a criterion for abnormality for the BCSB, finally classified as: (0) normal or (1) abnormal.

##### Verbal Fluency Test in the Animal Category

To evaluate verbal fluency, participants were asked to name as many animals as they could in one minute. The individuals received the following instructions: “*you must say the animal names you remember, as quickly as possible. Any animal will do, four-legged, fish, birds, the more you say, the better*”. The cutoff for verbal fluency test normality was ≥9. This test was applied as part of the BCSB, as an interference factor.

##### Stick Design Test

To evaluate logical thinking, participants were asked to reproduce geometric shapes, presented on a sheet of paper one after the other, with matches. The number of sides, the image and stick orientation in each of the four images were criteria considered for the test score, which varies from zero to 12. Scores ≥10 were considered the cutoff for normality and classified as normal (0) or abnormal (1).

### 2.4. MeHg Exposure Biomarker

According to the guidance published by the World Health Organization [56], and described by other authors [57,58], hair samples are considered the best biomarker of the MeHg human exposure, because almost all Hg detected in this kind of biological matrix is in the MeHg form. In addition, because the main Hg exposure route observed in the studied population is the consumption of Hg-contaminated fish, and almost all Hg present in the fish muscle is in the MeHg form, hair samples were used as its exposure biomarker [47,48,49]. Therefore, we assume for this study that all Hg present in hair samples is MeHg. 

The hair’s samples were collected from the occipital region, with the aid of stainless-steel dissection scissors, from all participants. The samples were stored in individually identified paper envelopes and sent for total mercury concentration (THg) analysis in the Toxicology Laboratory in the Environmental Section of the *Instituto Evandro Chagas* (IEC). 

In the laboratory, before starting the analyses, hair samples were repeatedly washed with Extran detergent (Merck KGaA, Darmstadt, Germany) to remove any exogenous contamination. After drying, the samples were finely homogenized in glass flasks before weighing. This methodology comprises chemical opening, wet digestion, and subsequent reduction with SnCl_2_ to quantify total Hg in a Cold Vapor Atomic Absorption Spectrometer (CVAAS). 

The protocols for Quality Assurance (QA)/Quality Control (QC) included the following parameters: (i) a method blank; (ii) a 6-point calibration curve (concentration ranging from 0.4 to 4 ng/g); (iii) the Human Hair Certified Reference Material (IAEA-86), whose average recovery rate was 101% (n = 8, recovery ranging from 83.4 to 106.6%) from the International Atomic Energy Agency; and (iv) the relative standard deviation (RSD) of 8.32%. Sample replicates (n = 10), whose RSD was 2.49%, were also randomly selected. The detection and quantification limits (LOD/LOQ) obtained were 0.0083 ng/mg and 0.027 ng/mg, respectively. 

For further detail on hair sample analysis, see Basta et al. [43].

### 2.5. Statistical Analysis

A descriptive analysis of the participants was undertaken according to sociodemographic variables: sex (female / male), age (12 to 19; 20 to 24, 25 to 29, and ≥30 years old) and village of residence (*Sawré Muybu*, *Poxo Muybu*, and *Sawré Aboy*), in contrast to the evaluated neurological parameters. 

To estimate the prevalence of exposure, in the numerator we included participants older than 12 who presented with methylmercury levels ≥10.0 µg/g. In the denominator, the population sampled for the study in the same age group was considered. The prevalence was presented for the three villages under study: *Sawré Muybu*, *Poxo Muybu*, and *Sawré Aboy*.

The main neurological abnormalities detected in the clinical exam were compared between two groups, according to the participants’ level of methylmercury exposure: ≥10.0 vs. <10.0 µg/g.

Because mercury levels do not show a normal distribution, the Kruskal–Wallis non-parametric test was used to evaluate differences in methylmercury levels in all comparisons that showed measures of central tendency (medians). The difference in proportion between variables was evaluated using Pearson’s chi-squared test.

We used a Poisson regression model with robust variance to estimate the association between alterations observed in the BCSB and the verbal fluency test in the animal category with independent variables: (i) methylmercury exposure level (<10.0 vs. ≥10.0 µg/g); age (12 to 19; 20 to 24, 25 to 29, and ≥30 years old); and (ii) sex (female vs. male). Prevalence Ratio (PR), with the respective confidence interval of 95%, was used as an association measure.

Variables with a *p*-value < 0.20 in the simple analysis were selected and included in the multiple model. Variables that presented significance levels of 5% (*p* < 0.05) remained in the final model. The data was analyzed with the statistical software SPSS version 9.0 (Chicago, IL, USA).

## 3. Results

During the field work, 110 indigenous individuals were evaluated, 50 of whom were from the *Sawré Muybu* (SM) village, 37 from the *Poxo Muybu* (PM) village, and 23 from the *Sawré Aboy* (SA) village. Fifty-eight were men (52.7%) and 52 were women (47.3%). The participants age ranged from 12 to 72 years (average: 27.6 years; standard deviation: 13.7). The participants’ distribution, according to age group, was heterogenous in the studied villages. There was a predominance of adolescents (12 to 19 years) in PM and SA villages, with 16 (43.2%) and 11 (47.8%) participants, respectively, compared to nine (18.0%) participants in the SM village. In contrast, in *Sawré Muybu* there was the highest concentration of young adults between 25 and 29 years of age (26.0%) (*p* = 0.032) (Table 1).

### 3.1. Overall Data by Village

No participant showed static balance, walking, or cranial nerve function alterations. There were significant somatosensory alterations in the SM and SA villages in contrast to PM, represented here by the distal pinprick perception (*p* = 0.012), distal thermal sensitivity (*p* = 0.004), hallux or thumb vibration sensitivity (pallesthesia) (*p* = 0.042), and feet mechanical detection threshold (*p* = −0.001). Clinical signs of polyneuropathy were detected in 21 (42.0%) participants from *Sawré Muybu* village and 10 (43.5%) participants from *Sawré Aboy* village, revealing a significant superior prevalence compared to *Poxo Muybu* village, where only 4 (10.8%) participants were affected (*p* = 0.003) (Table 1).

Two (1.9%) indigenous people presented with alterations in cerebellar motor alterations ataxy, one from SM and one from SA villages, and three (2.8%) presented with bradykinesia, two of which were from SA and one from SM village, with no significant differences in the distribution between the villages. No signs of hypertonia or rigidity were detected in participants (Table 1).

Five (4.5%) participants were diagnosed with toe amyotrophy, three of which were from PM village and two from SM village (*p*-value = 0.331). There were no recorded cases of amyotrophy in SA.

The absence of ankle jerk reflex was detected in 10 (20%) and six (26.1%) participants from SM and SA village, respectively, and was more prevalent than in PM village, where it was observed in only one (2.7%) individual (*p* = 0.025) (Table 1).

Regarding cognitive manifestations, there were BCSB abnormalities in 39.1% (n = 9) of participants from SA village, whereas in SM and PM the alterations affected 17.6% (n = 9) and 21.6% (n = 8) of participants, respectively (*p* = 0.089) (Table 1). 

Impairments in the verbal fluency test were detected in 21.6% (n = 6) of participants from SA village. In contrast, in PM and SM, the verbal fluency affected 10.8% (n = 4) and 6.0% (n = 3) of participants, respectively (*p* = 0.046) (Table 1).

Alterations in the stick design test were observed in only one (2.0%) individual from SM village and in one (4.3%) individual from SA (*p* = 0.466).

### 3.2. Neurological Abnormalities According to MeHg Exposure Levels

In general, the median MeHg level in participants was 7.4 µg/g (average: 8.7; standard deviation: 4.5; range from 2.0 to 22.8). In *Poxo Muybu* village, the median MeHg level in participants was 7.3 µg/g (average: 7.3; standard deviation: 2.3; range from 2.3 to 12.9). In *Sawré Muybu* village, the median MeHg level in participants was 6.5 µg/g (average: 7.4; standard deviation: 4.2; range from 2.0 to 22.1). Finally, in *Sawré Aboy* village, the median MeHg level in participants was 12.5 µg/g (average: 13.5; standard deviation: 4.6; range from 4.8 to 22.8), showing a significant statistical exposure gradient (Kruskal–Wallis Test with *p*-value < 0.001).

The MeHg exposure prevalence over 10 µg/g also varied according to the studied villages, 78.3% of which from SA, 22.4% from SM, and 10.8% from PM (*p*-value = 0.001) (Table 1). However, there were no significant differences in MeHg exposure considering age group and sex (Table 2).

#### 3.2.1. Somatic Sensitivity (Somatosensory)

Alterations in distal pinprick perception were found in 14.5 and 12.1% of participants in the exposure groups of <10 and ≥10 µg/g MeHg, respectively (*p*-value = 0.743) (Table 2). 

Distal thermal sensitivity alterations were observed in 14.5% of participants of exposure group of <10 µg/g, in comparison to 21.2% in the group of ≥10 µg/g MeHg (*p*-value = 0.384) (Table 2). 

Hallux or thumb vibration sensitivity alterations were seen in 6.6% of participants in the exposure group of <10 µg/g, in contrast to 15.2% in the exposure group of ≥10 µg/g MeHg (*p* = 0.154) (Table 2).

Feet mechanical detection threshold (hypoesthesia) alterations were detected in 18.4% of participants in the exposure group of <10 µg/g, in contrast to 33.3% in the exposure group of ≥10 µg/g MeHg (*p* = 0.089) (Table 2).

Finally, the clinical signs of polyneuropathy, indicative of distal symmetric polyneuropathy, were similar in the groups, and were reported in 30.3% of indigenous individuals with the exposure <10 µg/g and in 36.4% of those with exposure ≥10 µg/g MeHg (*p* = 0.531) (Table 2).

#### 3.2.2. Motor Function

Toe amyotrophy was recorded in five individuals (6.6%) in SM village. The ankle jerk reflex absence appeared in 13.2 and 21.2% of indigenous individuals exposed to levels of MeHg <10 µg/g and ≥10.0 µg/g (*p* = 0.287), respectively (Table 2). 

The occurrence of bradykinesia was detected in three indigenous individuals: an individual with a methylmercury exposure level of 9.08 µg/g, and in two other participants with exposure levels of 11.68 and 20.18 µg/g (*p* = 0.164) (Table 2).

Cerebellar motor incoordination (ataxia) was observed in two participants with exposure levels of 11.68 and 15.86 µg/g MeHg (*p* = 0.030).

#### 3.2.3. Cognitive Alterations 

According to the BCSB, cognitive alterations were observed in 17.1% of indigenous individuals with MeHg exposure levels of <10 µg/g, in contrast with 36.4% of those with an exposure of ≥10 µg/g (*p* = 0.028) (Table 2). 

Moreover, the participants with MeHg exposure levels of <10 µg/g showed 7.9% of alteration in the verbal fluency test in the animal category, whereas 21.2% of participants with MeHg exposure levels of ≥10 µg/g presented alterations (*p* = 0.049) (Table 2).

Two individuals (1.8% of the total) presented with alterations in the stick design test, one with a MeHg exposure level of 9.67 µg/g, and the other with 22.75 µg/g (Table 2). It is worth noting that the recorded exposure level in the latter was the highest in the studied population.

#### 3.2.4. Main Findings Considering the Neurological Abnormalities, According to MeHg Exposure Levels

Table 3 summarizes the main results, highlighting neurological clinical variables that presented an association with hair-MeHg levels (≥10 µg/g). Considering the presence of somatosensory signs, abnormalities in the feet mechanical detection threshold affected 33.3% of the participants with MeHg levels above 10 µg/g, in contrast to 18.4% of the participants with MeHg levels below 10 µg/g. Regarding motor function, only two cases of ataxia were detected. However, both were observed in participants with MeHg levels above 10 µg/g. 

Regarding the cognition abilities, 36.4% of the participants with MeHg levels above 10 µg/g presented abnormalities in the Brief Cognitive Screening Battery (BCSB). In contrast, 17.1% of the participants with MeHg levels below 10 µg/g showed abnormalities in the BCSB. Finally, concerning the verbal fluency test, 21.2% of the participants with MeHg levels above 10 µg/g presented abnormalities, in contrast to 7.9% of the participants with MeHg levels below 10 µg/g (Table 3).

#### 3.2.5. Poisson’s Regression Model Findings, Considering Cognition Abilities of the Participants

Poisson’s regression modeling revealed that participants with MeHg exposure levels ≥10 µg/g showed approximately two-fold higher chances of suffering from cognitive deficits in the BCSB (PR: 2.2; CI 95%: 1.13–4.26) and the verbal fluency test (PR: 2.0; CI 95%: 1.18–3.35). Furthermore, the final model showed that 12 to 19 year old adolescents presented with around a three-fold chance of developing deficits in the verbal fluency test (PR: 3.2; CI 95%: 1.06–9.42), compared to 20 to 24 year old young adults, even controlling for the effect of mercury exposure level and sex (Table 4).

## 4. Discussion

This exploratory clinical-epidemiological study was the first to produce evidence about the neurotoxic effects of consuming contaminated fish by MeHg in adults from the Munduruku indigenous population. The findings were produced with a comparative analysis of systemized neurological evaluation data in contrast to hair-MeHg concentration, used as a biomarker of chronic mercury exposure.

There is strong evidence substantiating MeHg neurotoxicity in experimental models and humans [33,34,35,42]. The neurological manifestations, classically described after the acute intoxication by MeHg, in Minamata Bay, Japan, in 1953, are characterized by somatosensory dysfunctions, ataxia, incoordination, tremors, and visual and neuropsychiatric symptoms [8,9]. A study conducted by Yorifuji et al. [59] also confirms that the neurological manifestations are due to the methylmercury contamination and are irrefutable evidence that the toxicity produced by this chemical element lasts for decades. 

However, in the context of chronic environmental exposure, there is still no solid causal connection between the neurological dysfunction and toxicity due to methylmercury, as highlighted by Puty et al. [60], who conducted a systematic review of this theme. Despite the relevance of this systematic review in consolidating the scientific knowledge, it is essential to emphasize that this study included only six papers, and most of these (i.e., four) do not provide data on mercury concentration in exposure biomarkers. Consequently, we can conclude that almost all selected research in that review presumptively reported human mercury exposure.

Nonetheless, when we focus only on research conducted in the Amazon region, we observe that most of these studies indicate that exposure to MeHg may be responsible for neurological alterations in adult individuals [13,14,15,16,17,18,19]. In this region, the mercury levels detected in exposure biomarkers can be up to 10-fold higher than the limits recommended by health agencies [45,46]. The main neurological alterations include cognitive, visual, motor, somatosensory, and behavioral changes [13,14,15,16,17,18,19,61,62,63,64,65,66,67,68].

The impact of gold mining on human mercury exposure has been described by some studies that compared exposed populations to control groups (for example, populations living in areas with no previous history of mining activities) [16,69,70,71,72]. These studies indicate that hair mercury levels detected in areas impacted by mining activities are considerably higher than those observed in control groups. Even considering the natural mercury available in the Amazon soil, these findings reinforce the hypothesis that the region’s primary source of human mercury contamination is mining activities. 

In the present study, somatosensory, motor, and cognitive abnormalities were researched in indigenous adults from the Munduruku people who live in three villages, located in the region of Middle-Tapajos River. The participants with higher mercury exposure levels (≥10 µg/g MeHg) presented the highest prevalence of cognitive impairment, according to the BCSB and the verbal fluency test results, and cerebellar incoordination, both indicating CNS dysfunctions. Distal somatosensory deficits and verbal fluency reduction were also reported. These alterations were more frequent in *Sawré Aboy* village, where there was the highest mercury exposure, compared to *Sawré Muybu* and *Poxo Muybu* villages. Again, this finding strengthens the hypothesis that the primary source of mercury exposure in the studied villages is mining activities.

### 4.1. Somatosensory Abnormalities

In the group with the highest MeHg exposure (≥10 µg/g), there was also a higher prevalence of abnormalities in the feet mechanical detection threshold, in comparison to the group with lower exposure (33.3% vs. 18.4%, *p* = 0.089). There was a slim predominance of temperature and vibrant hypoesthesia in participants with methylmercury exposure higher than 10 µg/g (Table 2), however, there was no statistical significance. Even considering different methodological approaches, our findings revealed the highest somatosensory abnormalities in comparison to the study conducted by Khoury et al. [63,64], in which there was a decrease in hypoesthesia, tactile sensitivity, and pain, in 11.5% (n = 9) of riparian residents in Barreiras and 13.3% (n = 4) of residents in São Luiz do Tapajós, areas in the Pará State, in the Amazon Basin, equally affected by mining activities. In turn, the somatosensory alterations reported here were higher than those observed in a region known as *Furo do Maracujá* (n = 1; 2.0%), an area considered free of mercury exposure, and therefore classified as a control area by the authors [63,64]. However, due to the small number of participants, the differences highlighted in the study were not statistically significant.

The comparison by village revealed a significantly lower prevalence of somatosensory abnormalities in *Poxo Muybu* village, in contrast to *Sawré Aboy* and *Sawré Muybu* villages (Table 1). The difference in occurrence of alterations was revealed in all sensory modalities investigated, either in the tactile superficial sensory (*p* = 0.001), temperature sensitivity (*p* = 0.004) and pain sensitivity (nociceptive) (*p* = 0.012), and hallux and thumb deep sensitivity (vibration) (*p* = 0.042). In the same manner, *Poxo Muybu* was the village where lower frequencies were reported of ankle jerk reflex alterations (*p* = 0.025) and clinical signs of polyneuropathy (*p* = 0.003).

These findings provide evidence of the toxic effects of methylmercury, because *Poxo Muybu* village recorded the lowest proportion of participants with exposure levels above 10 µg/g MeHg (10.8%), whereas in *Sawré Muybu* and *Sawré Aboy* villages the exposure prevalence was approximately two to eight times higher, reaching 22.4% and 78.3% of participants, respectively.

However, although there is a tendency of major abnormalities in distal thermal sensory, deep vibration, and tactile superficial sensitivity, the magnitude of the somatosensory alterations observed in the comparative analysis by village was not confirmed, according to the methylmercury exposure groups. In part, this contradiction can be explained by the reduced size of our sample. However, it is important to note that the average methylmercury levels in all participants were high (average: 8.7 µg/g; range from 2.0 to 22.8), which makes it difficult for more robust analysis, because we did not have a control group, without mercury exposure, for comparison.

It is important to note that distal somatosensory alterations and clinical signs of polyneuropathy are indicative of peripheral nervous system (PNS) dysfunctions that can be induced by several clinical conditions in addition to mercury contamination. The presence of chronic alcoholism, diabetes mellitus, and sexually transmittable diseases was actively researched in our study population, via interview, blood glucose level testing, or rapid HIV, syphilis, and viral hepatitis testing, with no positive cases identified (for further detail see Basta et al. [43]). Nutritional deficits, especially B12 deficiency, are also potential causes of polyneuropathy and somatosensory abnormalities. However, these nutritional deficiencies were not researched and may add a degree of bias to our findings. It must also be noted that the use of a hair sample as a biomarker for mercury exposure may not be adequate, especially in the adult population, for detecting the neuropathy cases [33]. It is possible that the clinical signs of neuropathy detected here may have begun prior to our research for reasons not evaluated.

However, it cannot by overlooked that the clinical signs of polyneuropathy, diagnosed in approximately one-third of all participants, were more frequent in *Sawré Muybu* and *Sawré Aboy* villages, where the highest levels of MeHg exposure were detected.

Similar to the results reported in this study, Takaoka et al. [73] compared an indigenous population in Canada who lived in a region affected by chloralkali industrial waste, with two populations in Japan, one exposed to mercury in Minamata Bay and the other in a non-affected area (control). The authors reported complaints and neurological abnormalities as more prevalent. In addition, somatosensory signs were more evident in the Canadian indigenous group and in the Japanese group exposed to mercury compared to the control group. 

In another study conducted in Japan, Takaoka et al. [38] presented a detailed description of somatosensory symptoms that included perioral numbness, hands and feet numbness, cramps, difficulty in hearing, smelling, and tasting, difficulty in buttoning shirts, and difficulty executing fine tasks with fingers. The authors reported that the symptoms were more frequently observed in individuals who ate fish with Hg concentrations higher than levels considered to be safe. They concluded that their findings indicated that the effects of Minamata disease were observed almost 50 years after Chisso Corporation stopped polluting water with methylmercury, in 1968.

In our study, the presence of distal hypoesthesia and clinical signs of polyneuropathy were respectively observed in 33.3% and 36.4% in the group with the highest mercury exposure (≥10 µg/g). These findings were similar to those reported by Yorifuri et al. [59], who showed somatosensory abnormalities in 25 and 58% of participants with mercury exposure higher than 10 µg/g in the contaminated areas of Minamata and Goshonoura, Japan, respectively.

In the Amazon region, Khoury et al. [63,64] described somatosensory complaints of numbness of the extremities in 11.5 and 13.3% of participants of riparian communities in two areas of the state of Pará, which are under the influence of mining and, therefore, exposed to mercury.

Due to communication difficulties arising because Munduruku people express themselves in their native language and do not speak fluent Portuguese, in this study, we exclusively evaluated clinical signs through a patterned neurological exam. In contrast to other authors [8,63,64,73], we opted not to include any complaints or symptoms reported by participants in the collected data. 

### 4.2. Motor Functions Abnormalities

Cerebellar signs were only detected in two indigenous individuals, who presented with methylmercury exposure higher than 10 µg/g. Similarly, Takaoka et al. [39] described alterations in the finger to nose test, which indicates cerebellar dysfunction, in 21 and 47% of two groups with high mercury exposure, in Japan. Cerebellar symptomatology was also described in other empirical studies [15,39,59] and in revision papers [8,60,74]. In turn, Khoury et al. [63,64] described altered walking in participants with high mercury exposure levels compared to the control group. However, there was no mention of cerebellar signs.

Bradykinesia or slowness of movement were identified in three indigenous individuals in our study. They did not present rigidity or tremors. Bradykinesia, usually found in rigid-hypokinetic or parkinsonian syndromes, is not part of the classical manifestation of acute mercury intoxication, as described in Japan [8,59].

Nonetheless, experimental evidence associates mercury toxicity with dopaminergic depletion in the CNS [40] and establishes a possible correlation with parkinsonian and neurodegenerative diseases [75], even considering the latency period after mercury exposure [76]. In this sense, we propose two possibilities: (i) bradykinesia is an isolated symptom, or (ii) it may be a part of an incomplete parkinsonian syndrome falling within the spectrum of neurological manifestations of mercury contamination in this indigenous population from the Amazon. To clarify this question, we suggest a longitudinal evaluation in those populations at risk of chronic methylmercury exposure, with a priority for those cases with bradykinesia and other neurological signs and symptoms. In this manner, we believe that it will be possible to broaden the knowledge on this subject, which is surrounded by controversy and uncertainty.

### 4.3. Cognitive Abnormalities

Cognitive evaluation in individuals with a low education level is a challenging task, especially in indigenous populations who live in diverse sociocultural and environmental contexts. Language and communication barriers, in addition to the lack of appropriate evaluation instruments, were some of the difficulties experienced by our team. When choosing the evaluation instruments, we selected those that allowed us to overcome communication barriers, had references to the local population’s everyday elements, and were easily applicable in the context of the fieldwork in the villages. The BCSB domains used in this study allowed the evaluation of immediate memory, verbal fluency, and belated memory. It is essential to consider years of schooling, a factor significantly related to some cognitive tests [77] but not related to the ability to solve everyday problems, as highlighted in our evaluation.

Despite the limitations, the proportion of higher impairment rates in the BCSB in participants with high methylmercury exposure levels (≥10 µg/g) was more than two-fold (36.4%) those observed (17.1%) in participants with lower exposure levels (<10 µg/g) (*p* = 0.028). Similarly, Yorifuji et al. [59], in an enquiry that analyzed the Minamata population in the early 1970s, revealed significantly more frequent intelligence limitations (RP 5.2, 95% CI 3.7–7.3) and behavior alterations (RP 4.4, 95% CI 2.9–6.7) in the group exposed to methylmercury, in contrast to the control group.

It is worth noting that the neuropsychiatric manifestations, including irritability, depression, social isolation, aggressiveness, or even psychotic cases (“*mad hatter disease*”) were described in association with acute mercury intoxication [74]. In a bibliography review, Jackson [74] concluded that methylmercury poisoning causes chronic neurological illness, mainly due to direct neural lesions in the brain, particularly involving the cerebral cortex and granular cells, and reinforces the importance of detailed clinical evaluation to assess the extent of neurological deficits in chronically exposed populations.

In the Amazon scenario, the neuropsychiatric manifestations are more frequently associated with occupational exposure and are reported in miners who burn amalgam to separate mercury from gold and inhale mercury’s toxic vapors. Considering environmental exposure through the consumption of contaminated fish, which is manifested chronically and insidiously, there are few studies that report cognitive deficiencies and neuropsychiatric manifestations. Khoury et al. [63,64] analyzed depression complaints and symptoms such as fear, sadness, and insomnia in the riparian population in two areas in the Amazon with different mercury exposure levels. Despite the effort made in the analysis, the authors found that there were no statistical differences between the exposed and control groups.

In our study, the detected memory and verbal fluency impairments indicate cognitive deficits are more frequent in the indigenous people with the highest methylmercury levels (≥10 µg/g). Moreover, cognitive impairment was more frequent in 12- to 19-year-old adolescents. Because chronic methylmercury exposure may cause cognitive, motor, and somatosensory losses, this finding requires caution in interpretation and warrants deeper analysis. The investigated communities, who live in a highly contaminated environment, at present, in addition to the future generations (women of childbearing age and babies), are under permanent threat. Our findings emphasize the need for new studies that deepen cognitive and psychiatric evaluation to generate even more robust evidence about the MeHg toxicity in vulnerable populations, and demand interventions from the authorities and social leadership.

### 4.4. Limitations

Bradykinesia, temperature sensitivity, tactile and deep distal alterations, and clinical signs of polyneuropathy were more prevalent in the group with methylmercury exposure higher than 10 µg/g. However, due to the limited size of our sample, the differences were not statistically significant. The broadening of the study to other areas with the inclusion of a greater number of participants may result in more conclusive data.

In this study, we prioritized the evaluation of somatosensory abnormalities aimed at detecting PNS lesions. Somatosensory alterations from CNS lesions were described in anatomo-clinical studies [39] and clinical trials [8]. The two-point discrimination test, graphesthesia, and stereognosis have the potential to evaluate central somatosensory dysfunctions and should also be considered in future studies.

The absence of a control group with low environmental MeHg exposure, which would allow comparisons, was also a limiting factor in our approach. In the preparatory phase of the study, we expected that *Poxo Muybu* village, which is farther from the mining zones, would be a control group for comparison. However, like the other villages, *Poxo Muybu* also presented high average levels of MeHg exposure (average: 7.3 µg/g; range from 2.3 to 12.9), above the safe levels according to international health agencies [45,46]. Therefore, it was not possible to have a control group.

It is also important to consider that the reference levels of mercury exposure vary between international health agencies, and do not necessarily reflect biological security parameters. The safety limits recommended are based on daily or weekly maximum intake parameters of MeHg. The intake criteria have a strong relationship with MeHg levels detected in blood and hair. Therefore, FAO/WHO [45] recommends maximum intake doses of 1.6 µg/kg (body weight)/week for more sensitive groups such as women of childbearing age and pregnant women, and 3.2 µg/kg bw/week for the general population. Thus, the reference intake limit doses considered to be safe, for those still not reported to have poisonous effects in human beings, correspond to mercury levels of 2.3 and 4.5 µg/g in hair samples, respectively [45].

Although our results may show promise in revealing higher cognitive deficits in participants with the highest methylmercury exposure levels, the BCSB has not yet been validated for use in traditional Amazonian populations. The use of simplified and validated instruments for evaluation of cognition in populations chronically exposed to mercury and ethnically differentiated can produce new evidence to consolidate the knowledge on this topic. Tools such as the mini-mental state examination [78], previously used in Amazonian populations [77], appear to be equally promising, and mainly associated with the BCSB.

## 5. Conclusions

The present study produced evidence concerning the association between motor and cognitive impairments in the CNS with chronic MeHg exposure among the Munduruku indigenous adults. The majority of people who suffered from neurological abnormalities presented MeHg exposure levels above 10 µg/g. 

Somatosensory abnormalities reported in the villages where the highest methylmercury exposures were registered also indicate toxicity signs in the PNS. However, the differences reported between the two exposure groups were not statistically significant. Due to the limited sample size, we believe it is necessary to conduct broader studies to deepen the knowledge about the neurotoxic effects of methylmercury in the traditional populations living in the Amazon Basin, and particularly indigenous populations.

In conclusion, despite its limitations, this study indicates that neurotoxicity due to chronic environmental methylmercury exposure in the Munduruku indigenous population provides evidence of the long-lasting persistent environmental impacts in the Amazon [20,21,22,23,24]. The context revealed here highlights the risk looming over the present and future generations, who represent a substantial social, ethnic, and culturally diverse community, and should be considered and treated as the country’s most significant wealth. 

Finally, we believe monitoring and follow-up measures are necessary for the vulnerable populations chronically exposed to methylmercury who live in the Amazon basin. Moreover, we consider it essential to establish a basic care protocol for contaminated people in the Brazilian Unified Health System (SUS) with the support of specialists.

## Figures and Tables

**Table 1:** Sociodemographic variables, hair-methylmercury level groups, somatosensory and motor functions, and cognitive alterations, according to the village of residence. *Sawré Muybu* Indigenous Land, Pará state, Brazilian Amazon, 2019.

	Villages								
Sociodemographic Variables	*Sawré Muybu*		*Poxo Muybu*		*Sawré Aboy*		Total		*p*-Value
Sex	n	%	n	%	n	%	n	%	
Female	25	50.0	17	45.9	10	43.5	52	47.3	0.857
Male	25	50.0	20	54.1	13	56.5	58	52.7	
Total	50		37		23		110		
Age range (years)									
12 to 19	9	18.0	16	43.2	11	47.8	36	32.7	0.032
20 to 24	11	22.0	6	16.2	3	13.0	20	18.2	
25 to 29	13	26.0	2	5.4	2	8.7	17	15.5	
≥30	17	34.0	13	35.1	7	30.4	37	33.6	
Total	50		37		23		110		
Hair-methylmercury levels									
≥10 µg/g	11	22.4	4	10.8	18	78.3	33	30.3	0.001
<10 µg/g	38	77.6	33	89.2	5	21.7	76	69.7	
Total	49		37		23		109		
Somatosensory signs									
Distal pinprick perception									
Normal	40	80.0	37	100.0	18	78.3	95	86.4	0.012
Abnormal	10	20.0	0	0.0	5	21.7	15	13.6	
Total	50		37		23		110		
Distal Thermal Sensitivity									
Normal	38	76.0	37	100.0	17	73.0	92	83.6	0.004
Abnormal	12	24.0	0	0.0	6	26.1	18	16.4	
Total	50		37		23		110		
Hallux or Thumb Vibration Sensitivity									
Normal	46	92.0	36	97.3	18	78.3	100	90.9	0.042
Abnormal	4	8.0	1	2.7	5	21.7	10	9.1	
Total	50		37		23		110		
Feet mechanical detection threshold									
Normal	33	66.0	37	100.0	15	65.2	85	77.3	0.001
Abnormal	17	34.0	0	0.0	8	34.8	25	22.7	
Total	50		37		23		110		
Clinical signs of polyneuropathy (Neuropathy burden)									
No	29	58.0	33	89.2	13	56.4	75	68.2	0.003
Yes	21	42.0	4	10.8	10	43.5	35	31.8	
Total	50		37		23		110		
Motor functions									
Toe amyotrophy									
Absent	48	96.0	34	91.9	23	100.0	105	95.5	0.331
Present	2	4.0	3	8.1	0	0.0	5	4.5	
Total	50		37		23		110		
Ankle jerk reflex									
Normal	40	80.0	36	97.3	17	73.9	92	83.6	0.025
Abnormal	10	20.0	1	2.7	6	26.1	17	16.4	
Total	50		37		23		110		
Bradykinesia									
Absent	49	98.0	37	100.0	21	91.3	107	97.3	0.121
Present	1	2.0	0	0.0	2	8.7	3	2.7	
Total	50		37		23		110		
Cerebellar Ataxia									
Absent	49	98.0	37	100.0	21	95.5	108	98.2	0.468
Present	1	2.0	0	0.0	1	4.5	2	1.8	
Total	50		37		22		110		
Cognition									
Brief Cognitive Screening Battery (BCSB)									
Normal	42	82.4	29	78.4	14	60.9	85	76.6	0.124
Abnormal	9	17.6	8	21.6	9	39.1	26	23.4	
Total	51		37		23		111		
Stick design test									
Normal	50	98.0	37	100.0	22	95.7	109	98.2	0.466
Abnormal	1	2.0	0	0.0	1	4.3	2	1.8	
Total	51		37		23		111		
Verbal Fluency test									
Normal	47	94.0	33	89.2	17	73.9	97	88.2	0.046
Abnormal	3	6.0	4	10.8	6	26.1	13	11.8	
Total	50		37		23		110		

**Table 2:** Sociodemographic variables, somatosensory and motor functions, and cognition signs, according to hair-MeHg levels (<10 vs. ≥10 µg/g), *Sawré Muybu* Indigenous Land, Pará state, Brazilian Amazon, 2019.

	Hair-MeHg Levels						
Sociodemographic Variables	<10.0 µg/g		≥10.0 µg/g		Total		
Sex	n	%	n	%	n	%	*p*-value
Female	36	47.4	16	48.5	52	47.7	0.915
Male	40	52.6	17	51.5	57	52.3	
Total	76		33		109		
Age range (years)							
12 to 19	26	34.2	10	30.3	36	33.0	0.774
20 to 24	12	15.8	8	24.2	20	18.3	
25 to 29	12	15.8	5	15.2	17	15.6	
≥30	26	34.2	10	30.3	36	33.0	
Total	76		33		109		
Somatosensory signs							
Distal pinprick perception							
Normal	65	85.5	29	87.9	94	86.2	0.743
Abnormal	11	14.5	4	12.1	15	13.8	
Total	76		33		109		
Distal Thermal Sensitivity							
Normal	65	85.5	26	78.8	91	83.5	0.384
Abnormal	11	14.5	7	21.2	18	16.5	
Total	76		33		109		
Hallux or Thumb Vibration Sensitivity							
Normal	71	93.4	28	84.8	99	90.8	0.154
Abnormal	5	6.6	5	15.2	10	9.2	
Total	76		33		109		
Feet mechanical detection threshold							
Normal	62	81.6	22	66.7	84	77.1	0.089
Abnormal	14	18.4	11	33.3	25	22.9	
Total	76		33		109		
Clinical signs of polyneuropathy (neuropathy burden)							
No	53	69.7	21	63.6	74	67.9	0.531
Yes	23	30.3	12	36.4	35	32.1	
Total	76		33		109		
Motor function							
Toe amyotrophy							
Absent	71	93.4	33	100.0	104	95.4	0.131
Present	5	6.6	0	0.0	5	4.6	
Total	76		33		109		
Ankle jerk reflex							
Normal	66	86.8	26	78.8	92	84.4	0.287
Abnormal	10	13.2	7	21.2	17	15.6	
Total	76		33		109		
Bradykinesia							
Absent	75	98.7	31	93.9	106	97.2	0.164
Present	1	1.3	2	6.1	3	2.8	
Total	76		33		109		
Cerebellar incoordination (ataxia)							
Absent	76	100.0	31	93.9	107	98.2	0.030
Present	0	0.0	2	6.1	2	1.8	
Total	76		33		109		
Cognition							
Brief Cognitive Screening Battery (BCSB)							
Normal	63	82.9	21	63.6	84	77.1	0.028
Abnormal	13	17.1	12	36.4	25	22.9	
Total	76		33		109		
Stick design test							
Normal	75	98.7	32	97.0	107	98.2	0.540
Abnormal	1	1.3	1	3.0	2	1.8	
Total	76		33		109		
Verbal Fluency test							
Normal	70	92.1	26	78.8	96	88.1	0.049
Abnormal	6	7.9	7	21.2	13	11.9	
Total	76		33		109		

**Table 3:** Summary of the main results highlighting clinical variables that presented an association (*p*-value < 0.10) with hair-MeHg levels (≥10 µg/g), *Sawré Muybu* Indigenous Land, Pará state, Brazilian Amazon, 2019.

	Hair-MeHg Levels						
Clinical Variables	<10.0 µg/g		≥10.0 µg/g		Total		
Somatosensory Signs	N	%	n	%	n	%	*p*-Value
Feet mechanical detection threshold							
Normal	62	81.6	22	66.7	84	77.1	0.089
Abnormal	14	18.4	11	33.3	25	22.9	
Total	76		33		109		
**Motor function**							
Cerebellar incoordination (ataxia)							
Absent	76	100.0	31	93.9	107	98.2	0.030
Present	0	0.0	2	6.1	2	1.8	
Total	76		33		109		
**Cognition**							
Brief Cognitive Screening Battery (BCSB)							
Normal	63	82.9	21	63.6	84	77.1	0.028
Abnormal	13	17.1	12	36.4	25	22.9	
Total	76		33		109		
Verbal Fluency test							
Normal	70	92.1	26	78.8	96	88.1	0.049
Abnormal	6	7.9	7	21.2	13	11.9	
Total	76		33		109		

**Table 4:** Logistic regression model (crude and adjusted) of prevalence ratios (PR) in the Brief Cognitive Screening Battery (BCSB) and verbal fluency test, *Sawré Muybu* Indigenous Land, Pará state, Brazilian Amazon, 2019.

Cognitive Screening Brief Battery				
**Variables**	**PR Crude (CI 95%)**	***p*-Value**	**PR Adjusted (CI 95%)**	***p*-Value**
Hair-MeHg level				
<10 µg/g	1.0		1.0	
≥10 µg/g	2.1 (1.08–4.15)	0.027	2.2 (1.13–4.26)	0.028
Age range (years)				
20 to 24	1.0		1.0	
12 to 19	1.3 (0.38–4.47)	0.681	1.5 (0.44–4.80)	0.424
25 to 29	1.7 (0.51–5.46)	0.399	1.9 (0.60–6.05)	0.280
≥30	2.2 (0.65–7.61)	0.204	2.4 (0.74–7.82)	0.144
**Sex**				
Female	1.0		1.0	
Male	1.1 (0.57–2.29)	0.71	1.2 (0.61–2.32)	0.492
**Verbal Fluency Test**				
**Variables**	**PR Crude (CI 95%)**	***p*** **-Value**	**PR Adjusted (CI 95%)**	***p*** **-Value**
Hair-MeHg level				
<10 µg/g	1.0		1.0	
≥10 µg/g	1.9 (1.07–3.38)	0.03	2.0 (1.18–3.35)	0.010
Age range (years)				
20 to 24	1.0		1.0	
12 to 19	3.0 (0.98–8.95)	0.054	3.2 (1.06–9.42)	0.039
25 to 29	0.8 (0.15–4.16)	0.775	0.9 (0.16–4.64)	0.871
≥30	1.8 (0.56–5.81)	0.324	2.0 (0.63–6.46)	0.239
Sex				
Female	1.0		1.0	
Male	0.6 (0.31–1.05)	0.071	0.6 (0.34–1.11)	0.109

## Data Availability

Data sharing not applicable.
